# Mechanistic insights into dimerization and cross-β sheet formation in the HIV-associated amyloidogenic peptide PAP248–286 from unbiased all-atom molecular dynamics simulations

**DOI:** 10.1016/j.csbj.2025.12.012

**Published:** 2025-12-16

**Authors:** Nikhil Agrawal, Emilio Parisini

**Affiliations:** a Latvian Institute of Organic Synthesis, Riga 1006, Latvia; b College of Health Sciences, University of KwaZulu-Natal, Durban 4000, South Africa; c Department of Chemistry “G. Ciamician”, University of Bologna, Bologna 40129, Italy

**Keywords:** Amyloid, PAP248-286, MD simulations, Umbrella Sampling, HIV

## Abstract

Semen-derived enhancer of viral infection (SEVI) fibrils, assembled from the peptide fragment PAP248–286, enhance HIV transmission by promoting viral attachment to host cells. However, the molecular basis of SEVI nucleation and early aggregation remains unclear. Here, we conducted 80 independent all-atom molecular dynamics (MD) simulations spanning a total of 40 μs, together with 100 independent steered MD and umbrella sampling runs, to explore the dimerization and dissociation of PAP248–286. Our results indicate that peptide association and β-sheet formation are governed by a cooperative interplay between hydrogen bonding and hydrophobic interactions. Residue-level analyses identified Arg10, Val17, Glu19, and Ile20 as key contributors to inter-peptide binding, consistent with steric zipper motifs described in other amyloid systems. Steered MD revealed mechanically resilient dimers with average rupture forces of ∼20 kcal/mol/Å and multi-barrier unbinding behavior. Umbrella sampling estimated a peptide dissociation free energy of ∼8.7 kcal/mol, highlighting a clear thermodynamic separation between bound and unbound states. Together, these findings suggest that small β-sheet nuclei in PAP248–286 dimers act as cooperative intermediates that seed the formation of full-length cross-β structures, providing atomistic insights into the earliest steps of SEVI fibril assembly.

## Graphical Abstract

**Figure:**
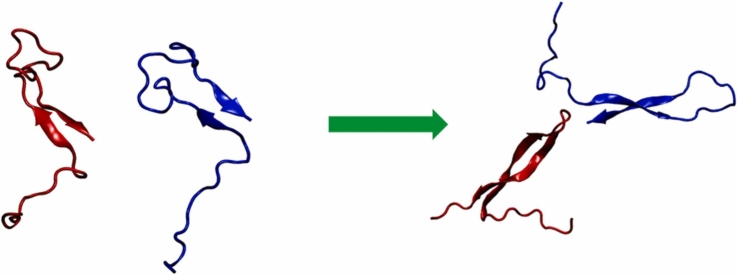

## 1 Introduction

Over the past four decades, HIV infection has emerged as a critical global health challenge. According to the World Health Organization, HIV has claimed an estimated 42.3 million lives worldwide. By the end of 2023, approximately 40 million individuals were living with HIV, including 20.5 million women [1], [2]. Unprotected sexual contact remains one of the primary routes of HIV transmission, with seminal fluid serving as a key vector for viral spread during sexual exposure [3], [4]. A significant factor contributing to this transmission is human prostatic acid phosphatase (PAP), a protein abundant in seminal fluid, which undergoes proteolytic cleavage to generate the peptide fragment PAP248–286 [5]. These peptides aggregate to form amyloid fibrils known as SEVI (Semen-derived Enhancer of Viral Infection). SEVI fibrils are highly cationic and promote viral attachment to mammalian cells. They have been reported to enhance HIV infection rates by up to 400,000-fold during sexual transmission [5], [6], [7], [8].

Given that amyloid formation is implicated in nearly 40 human diseases [9], [10], understanding the structural properties of SEVI fibrils is crucial. Despite sequence variations among amyloidogenic proteins, amyloid fibrils commonly adopt a cross β-sheet-rich structure [11]. The fibrillation process involves a series of structural transitions, including intermediate states such as oligomers and protofibrils[12]. Among these intermediates, structured oligomers containing cross β-sheet motifs act as nucleation centers for mature fibril formation [13], [14]. Notably, in neurodegenerative disorders such as Alzheimer's disease, oligomers are often regarded as the most toxic species [15]. Similarly, a study by Münch et al. [5]. confirmed that SEVI amyloids adopt a cross β-sheet structure, while Usmani et al. [16], using atomic force microscopy (AFM), further demonstrated that SEVI forms a dense fibrillar network in human semen, with individual fibrils measuring 0.5–2 μm in length and 5–20 nm in diameter—closely resembling those formed *in vitro.* Understanding how these fibrils assemble is critically important, as it provides insights into the molecular mechanisms underlying SEVI’s function and its ability to enhance viral infection. Despite these advances, experimental structural information is currently limited to shorter SEVI fragment [17], and detailed structural characterization of the full-length PAP248–286 peptide, particularly at oligomeric and early aggregation stages, remains unavailable. In this context, MD simulations can play a pivotal role by modeling the self-assembly process at atomic detail, predicting transient intermediates, and offering structural insights that are difficult to capture experimentally. This integrative approach can bridge existing gaps in our understanding of SEVI’s amyloid architecture and functional behaviour.

Dimer formation is a critical step in amyloidogenesis, acting as a nucleation point for higher-order aggregation. Amyloid formation is believed to follow a pathway common to many amyloidogenic proteins, where the transition from monomers to dimers represents an early and essential stage of fibril assembly. Literature reports suggest that dimerization of amyloidogenic peptides is driven by intermolecular hydrogen bonding and hydrophobic interactions, facilitating β-sheet stabilization and the subsequent formation of higher-order oligomers [10], [11]. Several studies indicate that amyloid dimers initially adopt loosely structured conformations, which gradually reorganize into stable β-sheet-rich intermediates [11], [12]. These findings align with general amyloidogenesis mechanisms, wherein dimers and oligomers act as metastable species governing fibril growth kinetics [18], [19]. Therefore, understanding dimerization of PAP248–286 peptides is important for uncovering structural transition pathways, which could help in developing inhibitors aimed at blocking SEVI-mediated pathological processes.

## 2 Methods

In the present study, we have performed all-atom MD simulations to understand dimer formation of PAP248–286 peptides in five different forms, based on different initial secondary structure content in PAP248–286 (Table 1).

**Table 1:** The five different simulation types performed in this study.

**Type**	**System Description**	**No. of simulations**	**Structure obtained**	**Structure**
I	Both monomers containing short β-sheet structures	40	Conformation of PAP248–286 obtained from previous simulation study [20]	
II	Both monomers containing full-length helix structures	10	Monomer structures taken from PDB ID: 2L77 [21], [22]	
III	One monomer in short helix form, the other in short β-sheet form	10	Both monomer conformations of PAP248–286 obtained from previous study [20]	
IV	One monomer in helix form, the other in cross β-sheet structure	10	Helical monomer from PDB ID: 2L77 [21], [22]; Cross β-sheet monomer taken from one of the Type I simulations	
V	One monomer in short β-sheet form, the other in cross β-sheet structure	10	Short β-sheet conformation from previous study [20]; Cross β-sheet structure taken from one of the Type I simulations	

In this study, we employed the NMR structure of PAP248–286 (PDB ID: 2L77, BMRB ID: 17346) together with conformations generated in our previous simulations [20], [21], [22]. At pH 4.2, the histidine residues His3 and His23 were protonated, yielding an overall peptide charge of + 8. Hydrogen atoms were subsequently added with the H+ + server [23]. The chosen protonation state (pH ∼4.2) represents mildly acidic conditions relevant to sexual transmission environments, including the vaginal tract and other mucosal surfaces. All molecular dynamics simulations were performed using the CHARMM36m force field [24], an updated version of CHARMM36 [25] optimized for intrinsically disordered proteins and peptides.

Five distinct systems were constructed for simulation. Type I consisted of two PAP248–286 monomers in short β-sheet conformations solvated in 10,207 water molecules with 8 Cl⁻ counter ions. Type II contained two monomers in fully helical conformations solvated in 14,013 water molecules with 8 Cl⁻ counter ions. Type III comprised one monomer in a short helix and the other in a short β-sheet conformation, solvated in 17,489 water molecules with 8 Cl⁻ counter ions. Type IV contained one monomer in helical form and the other in a cross β-sheet structure, solvated in 11,265 water molecules with 8 Cl⁻ counter ions. Finally, Type V contained one monomer in short β-sheet and the other in cross β-sheet form, solvated in 19,641 water molecules with 8 Cl⁻ counter ions.

All systems were subjected to energy minimization using the steepest descent algorithm [26], followed by two equilibration phases of 500 ps each under NVT and NPT ensembles. During minimization and the first equilibration phase, positional restraints of 400 kJ mol⁻¹ nm⁻² were applied to backbone atoms and 40 kJ mol⁻¹ nm⁻² to sidechain atoms. In the second equilibration stage, restraints were applied only to backbone atoms. Production simulations were subsequently performed without restraints. Bond constraints for proteins were applied using the LINCS algorithm [27], and water geometries were maintained with the SETTLE algorithm [28]. Long-range electrostatics were treated using the Particle Mesh Ewald (PME) method [29], while van der Waals and short-range electrostatic interactions were truncated at 12 Å. The Nose–Hoover thermostat [30] and Parrinello–Rahman barostat [31] were used to maintain temperature and pressure at 310.15 K and 1 bar, respectively, each with a coupling constant of 1 ps. Integration of the equations of motion was performed with the leap-frog algorithm [32].

For the Type I system, 40 independent production simulations were performed, while 10 simulations were conducted for each of the remaining systems. Each trajectory was 500 ns in length, yielding a total of 80 simulations and an aggregate sampling time of 40 μs. Independent replicates were initiated from randomized velocity seeds. All simulations were carried out using GROMACS [33].

### 2.1 Steered molecular dynamics (SMD) simulations, umbrella sampling, and potential of mean force (PMF) calculations

To probe the mechanical stability of PAP248–286 dimers, SMD simulations were performed. A representative dimer conformation with the maximum number of inter-peptide hydrogen bonds was selected as the starting structure. One monomer was fixed, and a constant-velocity pulling force was applied to the center of mass of the second monomer along a defined reaction coordinate. A total of 100 independent SMD trajectories were generated and the maximum dissociation force (Fₘₐₓ), dissociation time (t_d), and displacement at rupture (d) were recorded for each trajectory. Force–time and displacement–time profiles were analyzed to characterize the multi-barrier unbinding process and assess the mechanical resilience of the β-sheet interface. To quantify the thermodynamic stability of the dimer, umbrella sampling was performed along the center-of-mass (COM) distance between the two peptides. Fifty-five windows spaced by 1.0 Å covered a COM distance range of 0–55 Å. Each window was equilibrated and sampled extensively, ensuring ≥ 20–30 % overlap between adjacent windows. Harmonic restraints maintained the COM separation in each window. The PMF was reconstructed using the weighted histogram analysis method (WHAM), and the binding free energy (ΔG_bind) was calculated as the free energy difference between the bound and unbound states, providing a quantitative measure of dimer stability. Statistical uncertainties in the PMF were estimated using the weighted histogram analysis method (WHAM) with 1000-step bootstrapping, providing confidence intervals for the free-energy profiles. For clustering analysis, frames in which the minimum distance between the two peptides was ≤ 3.5 Å were extracted from all 40 independent trajectories of the Type-I system. For the Type-II to Type-V systems, the same distance criterion was applied to all 10 independent trajectories for each system. Due to the large number of extracted frames for the Type-I system, frames were further subsampled at 50 ps intervals prior to clustering. Clustering of dimer conformations was performed using the BitClust program [34]. A RMSD cutoff of 12 Å was applied for the Type-I system, while a cutoff of 10 Å was used for the Type-II to Type-V systems, for clustering backbone atoms were used. For the Type-II to Type-V systems, all extracted frames satisfying the distance criterion were included in the clustering analysis without additional subsampling.

### 2.2 Analysis

Hydrogen bonds and minimum inter-peptide distances were calculated using the GROMACS [33] tools gmx hbond and gmx mindistance, respectively. Binding free energies and per-residue contributions were evaluated using the gmx_MMPBSA tool [35]. In the SMD simulations, force–distance profiles were analyzed to identify rupture events and characterize the strength and dynamics of peptide–peptide interactions. For umbrella sampling simulations, the WHAM was used to compute the PMF profiles along the chosen reaction coordinates.

## 3 Results

### 3.1 Interaction between two PAP248-286 monomers and change in conformation

Hydrogen bonding play a key role in protein–protein interactions, particularly in amyloid-forming systems, where it stabilizes extended β-sheet structures and facilitates the assembly of ordered aggregates [36]. Previous studies have indicated that inter-peptide distances below ∼6 Å are required to enable backbone–backbone hydrogen bonding [37], which is critical for maintaining β-sheet conformations and forming the cross-β spines characteristic of amyloid fibrils. In our study, across 40 independent simulations of the Type-I set, we observed multiple trajectories showing an increase in β-sheet content in both peptides upon interaction. Notably, this effect was unique to the Type-I simulations, as the other sets did not exhibit full cross β-sheet formation during peptide association (Supplementary Fig. 3–6). Consequently, our analysis focuses primarily on the Type-I data.

**Fig. 1:**
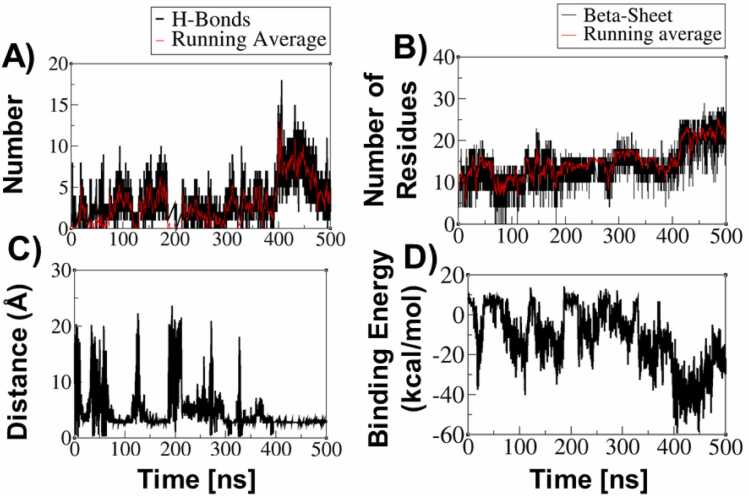
A) Time evolution of the number of H-Bonds, B) Time evolution of the number of residues of PAP248–286 monomers in β-sheet conformation, C) Minimum distance between two PAP248–286 monomers, and D) Time evolution of the binding energy between two PAP248–286 monomers.

**Fig. 2:**
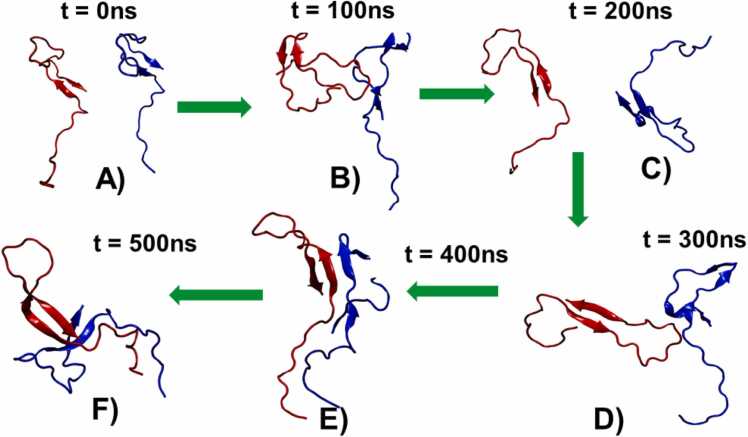
PAP248–286 monomer structures from one of the representative trajectory at six different time points, A) t = 0 ns, B) t = 100 ns, C) t = 200 ns, D) t = 300 ns, E) t = 400 ns, and F) t = 500 ns.

Fig. 1 presents the time evolution of hydrogen bonds, β-sheet content; inter monomer distance, and binding energy. In a representative trajectory, the minimum distance between the two monomers dropped below 5 Å around 400 ns (Fig. 1C), this distance, coinciding with a sharp increase in the number of hydrogen bonds formed between the peptides (Fig. 1A). This event was accompanied by a simultaneous rise in the number of residues adopting β-sheet conformations (Fig. 1B), reflecting enhanced structural ordering. Consistently, binding energy calculations (Fig. 1D) indicated a highly favorable interaction during the 400–450 ns interval, suggesting the establishment of a stable binding event. Fig. 2 illustrates the evolution of secondary structure content in the PAP248–286 monomers at different time points, highlighting the structural rearrangements induced by peptide association**.**

Overall, these findings suggest that the close approach of peptide monomers facilitates the formation of hydrogen bonds, which play a pivotal role in stabilizing β-sheet structures. This stabilization promotes the nucleation and growth of amyloid assemblies by driving the formation of stable, ordered aggregates, providing molecular-level insight into the early stages of amyloid fibril formation.

### 3.2 Identification of residues involved in the binding of PAP248-286 monomers

Several studies have shown that specific interactions between residues of one monomer and those of another play a central role in amyloid peptide assembly and promote β-sheet formation and stacking [9], [38]. In bacterial amyloids such as CsgA and TasA, conserved segments like LNIYQY and VTQVGF mediate cross-β interactions, where residues from one peptide align precisely with those from another, forming tightly interdigitated steric zippers [39], [40].

**Fig. 3:**
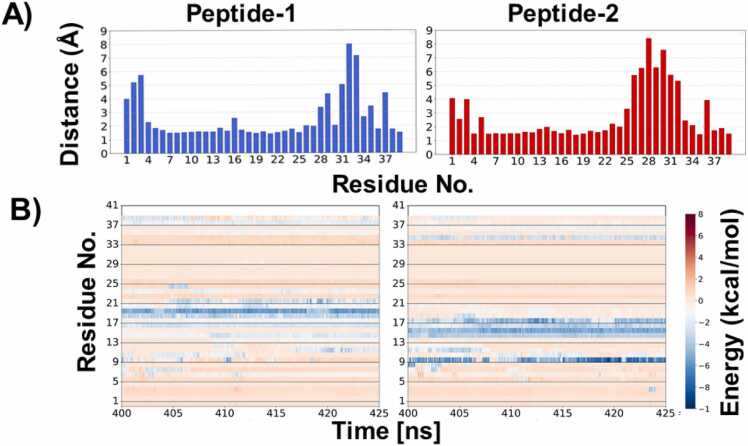
A) Minimum distance between both peptide during 400–425 ns, B) Time evolutions of contribution of each residue in binding.

To identify the residues and regions of each monomer involved in binding, we calculated the average minimum per residue inter-peptide distances during the 400–425 ns window—when binding energies were most favorable (Fig. 3A). Additionally, we computed the time evolution of the per-residue binding energy contributions during this period. The per-residue distance plot (Fig. 3A) revealed that residues 6–24 from both peptides were in closest proximity during this time.

To further pinpoint key contributors to binding, we analyzed the per-residue binding energy contributions between 400 and 425 ns (Fig. 3B). In the first peptide, residues 10–21 showed the highest contributions, while in the second peptide, residues 10–18 were most energetically involved. Among these, Glu19 and Ile20 from the first peptide, and Arg10, Gly14, Val15, Leu16, Val17, and Asn18 from the second peptide, played prominent roles in the interaction. These residues engage in both hydrophobic and electrostatic interactions, highlighting the importance of both types of forces in stabilizing the peptide-peptide interface.

### 3.3 SMD Simulations force and peptide dissociation

**Fig. 4:**
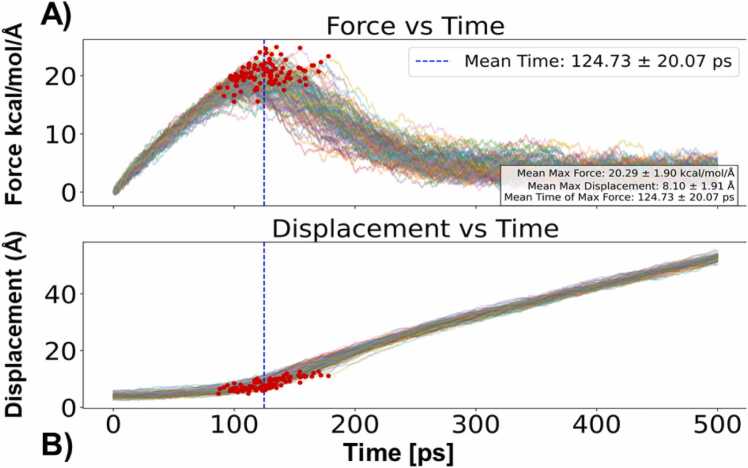
A) force vs time graph for all 100 SMD simulations, B) displacement vs time for 100 SMD simulations.

To investigate the strength of association between two monomers, we examined the unbinding mechanisms of two PAP248–286 monomers. We conducted 100 independent SMD simulations, applying constant-velocity pulling to dissociate spontaneously associated peptide dimers (Fig. 4). For the SMD simulations, we selected a starting conformation of the PAP248–286 dimer that contained the most inter peptide hydrogen bonds. From SMD simulations, we extracted three key descriptors: the maximum dissociation force (Fₘₐₓ), the dissociation time (t_d), and the displacement at unbinding (d). The force–time profiles (Fig. 4A) exhibited a characteristic pattern in which the pulling force gradually increased, peaking at an average Fₘₐₓ of 20.29 ± 1.90 kcal/mol/Å, before abruptly dropping at the dissociation event. This magnitude of force reflects the mechanical stability of the peptide complex, underscoring the strength of β-sheet-mediated inter-peptide interactions. Such mechanical robustness is consistent with previous AFM studies on amyloid fibrils, which have shown that β-sheet-rich aggregates resist dissociation forces in the nanonewton (nN) range [41], [42], and highlights the biological relevance of these structures in maintaining amyloid integrity under stress. The mean dissociation time (t_d) was found to be 124.73 ± 20.07 ps, with noticeable variability across trajectories, suggesting a rugged energy landscape with multiple intermediate states or alternative dissociation pathways. This observation aligns with earlier computational findings that amyloid peptide dissociation does not follow a single-path mechanism, but rather involves the crossing of multiple kinetic barriers. Furthermore, the displacement-time profiles (Fig. 4B) revealed an initial elastic extension of the complex followed by a sharp increase in displacement, marking the rupture point. The average displacement at the moment of dissociation was 8.10 ± 1.91 Å, corresponding to the disruption of backbone hydrogen bonds and side-chain contacts that stabilize the β-sheet interface.

Importantly, we observed a strong positive correlation (R² > 0.85) between Fₘₐₓ and t_d, indicating that complexes requiring higher dissociation forces also took longer to separate. This suggests a force-dependent, multi-step unbinding mechanism in which higher-energy barriers delay dissociation. Such behavior reinforces the notion that amyloid dissociation is governed more by kinetic control than by a single thermodynamic transition state. This is particularly significant given the role of β-sheets conformation in various physiological and pathological contexts, such as biofilm formation in bacteria or the persistence of neurotoxic aggregates in neurodegenerative diseases. The mechanical resistance of these assemblies, as captured in our SMD simulations, provides molecular insight into the interaction strength between two monomers of PAP248–286 in β-sheet conformation.

**Fig. 5:**
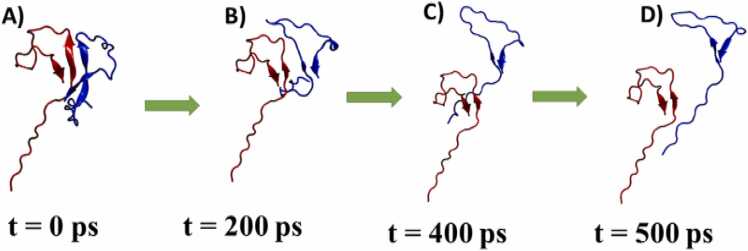
A) Snapshots of PAP248–286 monomer dissociation from one of the SMD trajectories, A) t = 0 ps, B) t = 200 ps, C) t = 400 ps, D) t = 500 ps.

Taken together, these results highlight the mechanically robust and kinetically complex nature of PAP248–286 peptides dissociation. The combination of force-resistance, displacement behavior, and variable dissociation timing underscores the cooperative nature of PAP248–286 monomer unbinding in β-sheet form as peptide dissociated β-sheet structure was reduced as appeared to be in conformation similar as it was at the beginning of the MD simulation (Fig. 5). By providing atomistic-level understanding of dissociation pathways, our study contributes to the broader understanding of amyloid stability, with potential implications for designing molecules that can modulate or disrupt pathogenic peptide aggregation.

### 3.4 Umbrella sampling and PMF analysis of peptide dissociation

To quantify the free energy profile associated with the dissociation of the peptide dimer, we performed umbrella-sampling simulations using a series of harmonic restraints along the center-of-mass (COM) distance between the two peptides. A total of 55 windows were generated with a spacing of 1.0 Å, covering a COM distance range from 0 to 55 Å. Each window was equilibrated and sampled extensively to ensure adequate overlap of the restrained configurations.

**Fig. 6:**
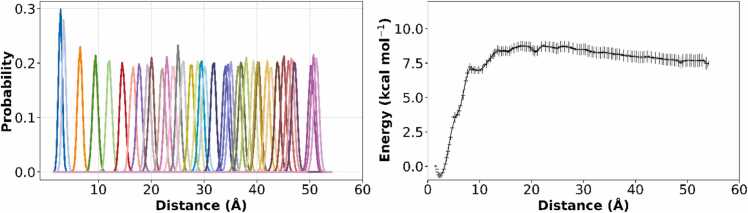
A) Probability distributions from each umbrella window, B) PMF profile indicating a bound state and unbound state of PAP248–286 monomers.

Fig. 6A illustrates the probability distribution of the reaction coordinate sampled in each umbrella window. The histograms demonstrate sufficient overlap between adjacent windows (≥ 20–30 %), indicating robust sampling across the entire reaction coordinate. This overlap is critical for reliable reconstruction of PMF via WHAM.

The resulting PMF curve is shown in Fig. 6B. A distinct global minimum is observed at short COM distances, corresponding to the bound state of the peptide dimer. The free energy gradually increases with separation, plateauing at larger distances that correspond to the unbound state. The bound state is located at approximately 3 Å with a free energy of ∼0.7 kcal/mol, while the unbound state stabilizes at ∼8.0 kcal/mol. The calculated dissociation free energy (ΔG_bind) is ∼8.7 kcal/mol, suggesting a moderately strong interaction between the two peptides in the dimeric state. This PMF profile provides critical insight into the thermodynamic stability of the dimer and supports the hypothesis that dimer formation is a key intermediate step during peptide aggregation.

### 3.5 Clustering of structures

Clustering of protein conformations from multiple molecular dynamics trajectories enables classification of structures into distinct ensembles that share similar topological features [43]. To characterize the dominant dimer conformations and assess their representativeness, we performed RMSD-based clustering of interacting PAP248–286 dimers extracted from all simulations (see Methods for details). Approximately 0.36 million structures were analyzed for the Type-I system, while for the Type-II, Type-III, and Type-IV systems approximately 0.42 million structures were included for each system, and for the Type-V system approximately 0.286 million structures were used. Only frames in which the minimum inter-peptide distance was ≤ 3.5 Å were considered to ensure that clustering focused on physically interacting dimer states. The clustering results reveal that the Type-I system is dominated by a β-sheet-rich dimer conformation (Supplementary Fig. 3), confirming that the representative structure shown in the main text corresponds to the most populated ensemble. In contrast, the Type-II to Type-IV systems display multiple dominant clusters with partial or asymmetric β-sheet organization and increased conformational variability, reflecting intermediate aggregation states (Supplementary Fig. 4–6). The Type-V system is characterized by more weakly associated and largely disordered dimers with limited β-sheet content (Supplementary Fig. 7). Together, these clustering analyses demonstrate that early PAP248–286 aggregation is structurally heterogeneous and system-dependent.

## 4 Discussion and conclusion

Our study provides an integrated view of the molecular determinants and energetics underlying the dimerization and dissociation of PAP248–286 peptides, a process central to SEVI fibril formation and its role in enhancing HIV infectivity. Through a combination of unbiased MD simulations residue-level interaction analysis, SMD, and umbrella sampling, we reveal how inter-peptide interactions drive conformational transitions critical to amyloid nucleation.

Our results demonstrate that PAP248–286 dimer stabilization arises from a cooperative interplay between hydrogen bonding and hydrophobic/van der Waals interactions. Hydrogen bonds primarily contribute to the directional alignment and stabilization of β-strands, while nonpolar side-chain contacts provide substantial energetic stabilization of the associated state. This balance of interactions facilitates the formation of β-sheet-rich dimers upon peptide association. This aligns with previous structural models of amyloid fibrils, where inter-strand hydrogen bonds and side-chain contacts form highly ordered, β-sheet-rich assemblies [36]. Notably, the residues identified as key contributors to binding—such as Glu19, Ile20, Arg10, and Val17—mirror interaction motifs observed in other amyloid systems like CsgA, which rely on steric zipper arrangements for stability [44], [45]. The mechanical stability of the dimer, as revealed by SMD simulations, is comparable to that observed in AFM studies of amyloids, with force–time and displacement–time profiles indicating a multi-barrier unbinding landscape [46], [47]. The observed strong correlation between unbinding force and dissociation time supports a kinetic model of dissociation, where rupture is governed not by a single transition state but by cooperative disruption of backbone and side-chain interactions—consistent with findings in other amyloid-forming system. Thermodynamic analysis via umbrella sampling corroborates these findings. The calculated dissociation free energy (∼8.7 kcal/mol) and clear separation between bound and unbound states confirm that dimer formation is a moderately favorable and distinct intermediate along the aggregation pathway. The significant histogram overlap further validates the robustness of the free energy estimation. Clustering analysis confirms the coexistence of β-sheet-rich and structurally heterogeneous dimers during early PAP248–286 aggregation. Within the 500 ns timescale of the unbiased MD simulations, we capture early aggregation processes such as peptide encounter, dimer formation, and local β-sheet organization, while later stages including higher-order oligomerization and fibril elongation are expected to occur on longer timescales. Overall, this work extends our previous monomer-level study by providing the first atomistic characterization of PAP248–286 dimerization and the key interactions stabilizing early aggregation intermediates. Collectively, these observations support a model in which PAP248–286 dimerization involves structurally and energetically cooperative interactions that stabilize β-sheet conformations. This structural transition, triggered by proximity and residue-specific contacts, likely serves as a nucleating event for higher-order aggregation. Given SEVI’s enhancement of HIV infection, these insights offer a molecular rationale for developing small molecules or peptides to disrupt early dimer formation and potentially modulate amyloid-related pathologies.

## CRediT authorship contribution statement

**Emilio Parisini:** Writing – review & editing, Conceptualization. **Nikhil Agrawal:** Writing – review & editing, Writing – original draft, Visualization, Validation, Software, Resources, Project administration, Methodology, Investigation, Funding acquisition, Formal analysis, Data curation, Conceptualization.

## Declaration of Competing Interest

The authors declare that they have no conflicts of interest related to this work.

## Data Availability

The PDB structures of the top five clusters for each system have been deposited in a public repository (Zenodo) and can be accessed at https://zenodo.org/records/17923514.
