# Supplementary material for: Mechanistic insights into dimerization and cross-β sheet formation in the HIV-associated amyloidogenic peptide PAP248–286 from unbiased all-atom molecular dynamics simulations
Source: Comput Struct Biotechnol J. 2025 Dec 16;31:74–81. doi: 10.1016/j.csbj.2025.12.012 (PMC12795694; doi:10.1016/j.csbj.2025.12.012)
Supplement: Supplementary file 1 — Supplementary material [file mmc1.docx]

**Mechanistic Insights into Dimerization and Cross-β Sheet Formation in the HIV-Associated Amyloidogenic Peptide PAP248–286 from Unbiased All-Atom Molecular Dynamics Simulations**

Nikhil Agrawal^1,2*^ and Emilio Parisini^1,3*^

^1^Latvian Institute of Organic Synthesis, Riga 1006, Latvia

^2^College of Health Sciences, University of KwaZulu-Natal, Durban 4000, South Africa

^3^Department of Chemistry “G. Ciamician”, University of Bologna, Bologna 40129, Italy

*Corresponding authors: [nikhil.08oct@gmail.com](mailto:nikhil.08oct@gmail.com) (Nikhil Agrawal), [emilio.parisini@osi.lv](mailto:emilio.parisini@osi.lv) (Emilio Parisini)


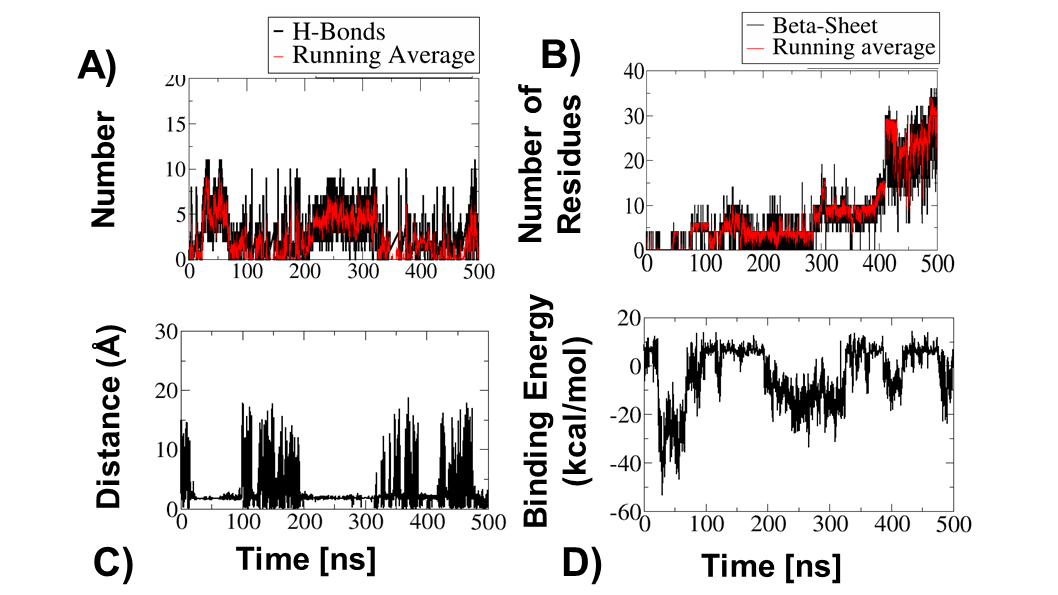


Supplementary Fig. 1 data from one of the respetivate trajectories of PAP248-286 from system type-I, A) Time evolution of the number of H-Bonds, B) Time evolution of the number of residues of PAP248-286 members in β-sheet conformation, C) Minimum distance between two PAP248-286 monomers, and D) Time evolution of the binding energy between two PAP248-286 monomers.


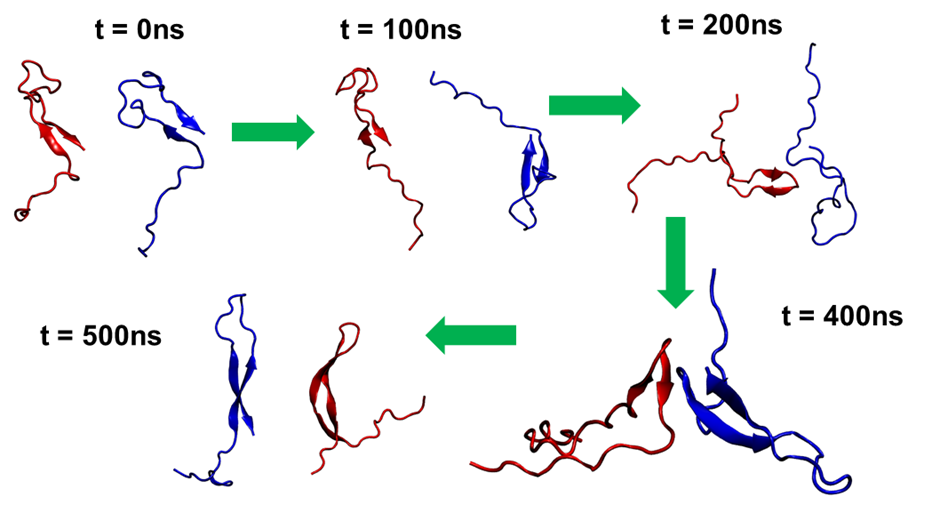


Supplementary Fig. 2. PAP248-286 monomer structures from one of the representative trajectory from system Type-I at five different time points, A) t = 0 ns, B) t = 100 ns, C) t = 200 ns, D, E) t = 400 ns, and F) t = 500 ns.


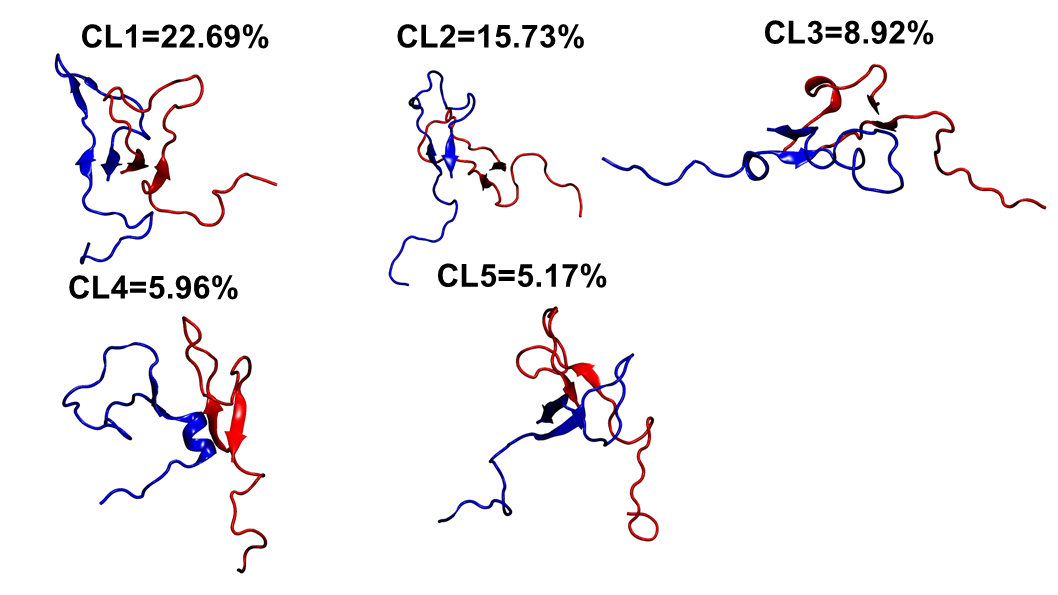


Supplementary Fig. 3 shows representative structures of the five most populated clusters identified by BitClust for the Type-I system.


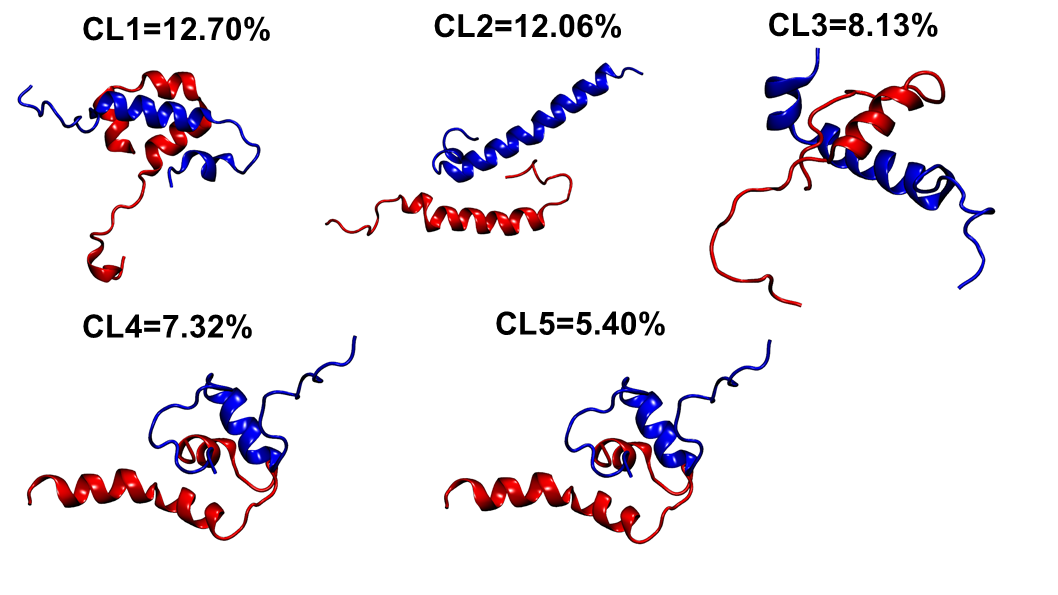


Supplementary Fig. 4 shows representative structures of the five most populated clusters identified by BitClust for the Type-II system.


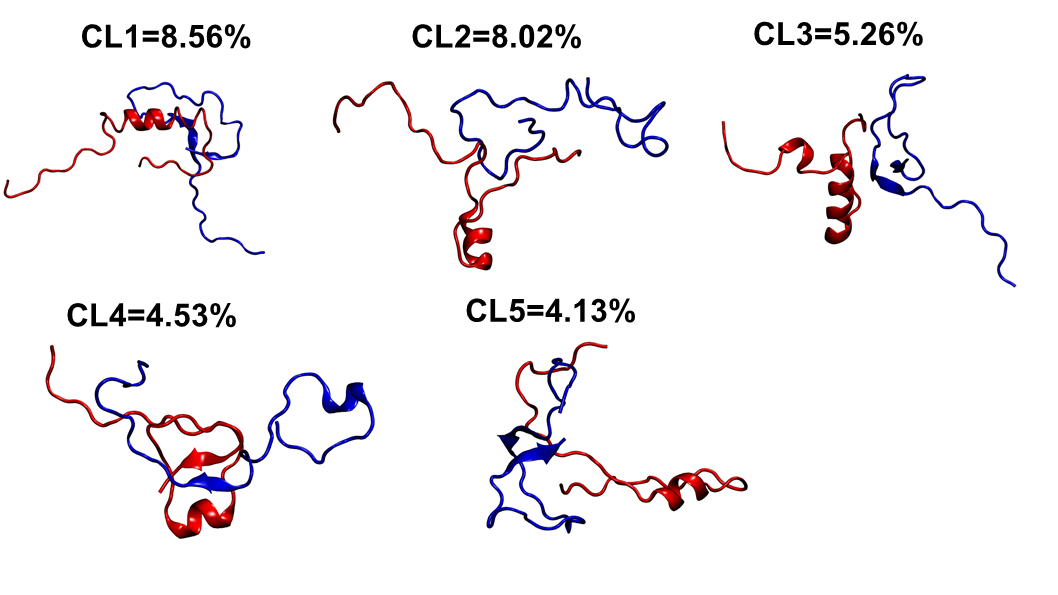


Supplementary Fig. 5 shows representative structures of the five most populated clusters identified by BitClust for the Type-III system.


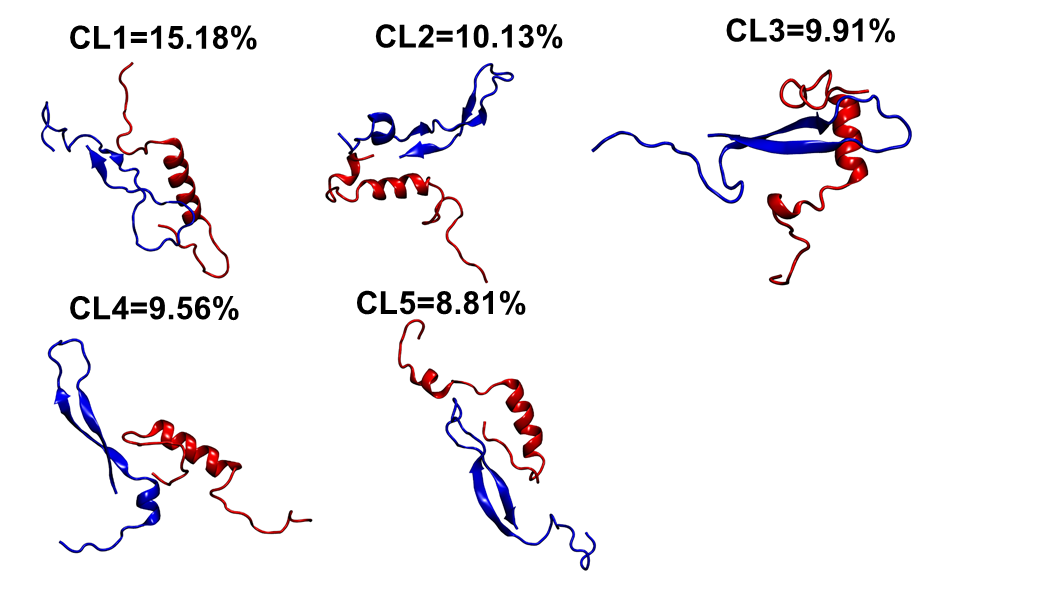


Supplementary Fig. 6 shows representative structures of the five most populated clusters identified by BitClust for the Type-IV system.


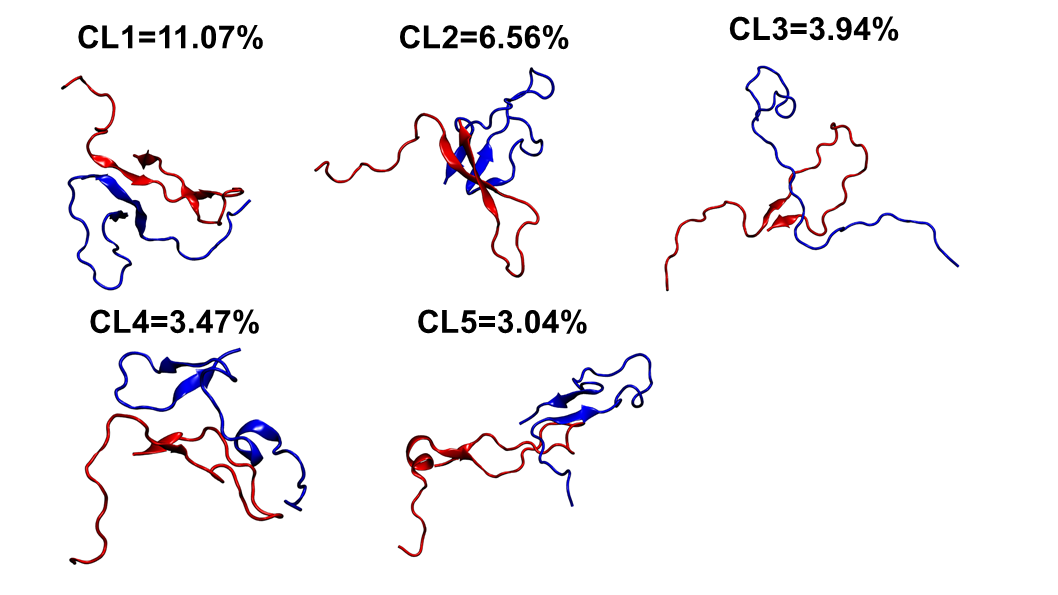


Supplementary Fig.7 shows representative structures of the five most populated clusters identified by BitClust for the Type-V system.
